# lncRNA POLR2J4 Plays a Biomarker Role in Hepatitis B Virus-Related Hepatocellular Carcinoma Through Regulating miR-214-3p

**DOI:** 10.5152/tjg.2024.24150

**Published:** 2024-10-01

**Authors:** Yimei Ji, Xiaowei Chen, Xin Liu, Jianyuan Huang, Pei Liu

**Affiliations:** 1 Department of Gastroenterology and Endoscopy, Third Affiliated Hospital of Naval Medical University, Shanghai, China; 2 Department of Interventional, The First Hospital of China Medical University, Shenyang, China; 3 Department of Infectious Diseases, The First People’s Hospital of Neijiang, Neijiang, China; 4 Department of General Surgery (Thyroid Gland/Blood Vessel), The First People’s Hospital of Neijiang, Neijiang, China; 5 Department of Ultrasound Interventional, Third Affiliated Hospital of Naval Medical University, Shanghai, China

**Keywords:** Hepatitis B virus, hepatocellular carcinoma, lncRNAs, POLR2J4, miR-214-3p, ceRNA, prognosis, development

## Abstract

**Background/Aims:**

Identifying novel therapeutic targets for hepatitis B virus (HBV)-induced hepatocellular carcinoma (HCC) has become a key goal in liver cancer research. Even though long non-coding RNAs (lncRNAs) do not code proteins, they could regulate the expression of functional genes and thus mediate disease development. The aim of this study was to estimate the role of lncRNA POLR2J4 (POLR2J4) in the progression of hepatitis B virus-related hepatocellular carcinoma (HBV–HCC) to pinpoint a potential biomarker.

**Materials and Methods:**

This study included 109 patients diagnosed with HBV-positive HCC, from whom tissue samples were collected. The expression level of POLR2J4 was evaluated by qPCR. The significance of POLR2J4 in HBV–HCC development and prognosis was estimated by Chi-square, Kaplan-Meier, and Cox analysis. In vitro, POLR2J4 was regulated in HBV–HCC cells, and its effect on cell growth and metastasis was assessed by CCK8 and Transwell assay. The interaction between POLR2J4 and miR-214-3p was evaluated through the luciferase reporter and RNA immunoprecipitation assays.

**Results:**

In tumor tissues of HBV–HCC patients, there was an observed increase in the expression of POLR2J4. The increase was closely related with patients’ presence of cirrhosis and vascular invasion, higher AFP, and advanced Edmondson grade and TNM stage. An upregulation of POLR2J4 predicted a poor prognosis for HBV–HCC patients and served as an independent indicator. In HBV-related HCC cells, silencing POLR2J4 suppressed cell proliferation, migration, and invasion. Furthermore, POLR2J4 negatively regulated miR-214-3p reversing the inhibition of cellular processes.

**Conclusion:**

POLR2J4 acted as a prognostic biomarker and a tumor promoter of HBV–HCC by modulating miR-214-3p.

**Main Points**

- Increasing POLR2J4 is closely associated with the malignancy of HBV–HCC.
- Increasing POLR2J4 indicates an adverse prognosis and high risk of HBV–HCC.
- POLR2J4 suppressed tumor progression by negatively modulating miR-214-3p.

## Introduction

Hepatocellular carcinoma (HCC), a major histological type of liver cancer, leads to global cancer-related incidence and deaths. Risk factors include chronic infection by hepatitis B virus (HBV) or hepatitis C virus (HCV), consumption of aflatoxins and nitrosamides, sustained intake of alcohol, non-alcoholic fatty liver disease, and steatohepatitis. These could induce cirrhosis, which could further develop into HCC.^1-4^ Despite the increasing commonality of hepatitis B vaccination, HBV infection still accounts for a significant portion of global liver cancer deaths.^1^ The prognosis for HCC patients varies greatly depending on the diagnosed stages. However, due to the insidious onset of HCC, the diagnosis of patients is mostly late. Current diagnostic and monitoring methods for HCC require supplemental indicators.^5,6^ The lack of sensitive and effective biomarkers, as well as concerns about malignant development, recurrence, and metastasis, complicate the clinical management of HCC, particularly for HBV-induced HCC.

In early research, protein-coding genes were considered the primary regulators of most biological processes.^7^ However, protein-coding sections of the genome constitute less than 2% of the total genome. This has led to growing interest in non-coding RNAs, including long non-coding RNAs (lncRNAs), miRNAs, and circRNAs. Long non-coding RNAs, in particular, have been recognized for their significant regulatory effect in human disease, influencing disease development and predicting patients’ outcomes.^8,9^ Recent studies identified potential biomarker lncRNAs in several human cancers, such as lncRNA HOTAIR in colorectal cancer, HOXA-AS3 in cervical cancer, and GAS8-AS1 in pancreatic cancer.^10-12^ In terms of HCC, several studies have established lncRNA signatures correlated with HCC development. Gu et al constructed a recurrence-free survival-related lncRNA signature for HBV-induced HCC, which consisted of 6 lncRNAs. Among these, lncRNA POLR2J4 showed great significance in the prognosis of hepatitis B virus-related hepatocellular carcinoma (HBV–HCC).^13^ Additionally, POLR2J4 was part of a 4-lncRNA signature linked to the overall survival of cirrhotic HCC and associated with severity-related features of patients.^14^ This suggests that POLR2J4 could serve as a potential biomarker for HBV-induced HCC. However, the specific function of POLR2J4 in HBV–HCC is not yet fully understood, and its status as a biomarker remains to be confirmed. This study thus aims to explore the expression and significance of POLR2J4 in HBV-induced HCC patients by analyzing clinical samples and data, with the goal of identifying a potential biomarker for HBV-induced HCC.

In mechanism, lncRNAs always sponge downstream miRNAs to display their function. Using an online database, miR-214-3p was predicted as a ceRNA of POLR2J4. miR-214-3p has been implicated in regulating cellular processes of HCC and is associated with patients’ prognosis.^15^ Additionally, miR-214-3p has been reported to mediate the enhancing effect of several lncRNAs, including LINC00665, HCG18, and HOXA11-AS on the development of HCC.^16-20^ With the employment of online databases, the target relationship between POLR2J4 and miR-214-3p was revealed with several binding sites. As such, miR-214-3p was hypothesized to be involved in the potential function of POLR2J4 in HBV-induced HCC. The biological effect and the potential molecular mechanism of POLR2J4 were assessed in HBV–HCC cells with the aim of uncovering the regulatory mechanism behind POLR2J4. This could potentially provide a target for the therapy of HBV-induced HCC.

## Materials and Methods

### Study Subjects

One hundred and nine patients were included from January 2014 to December 2016 based on the following criteria: (1) patients meet the diagnostic criteria of HCC and chronic hepatitis B; (2) the HBV surface antigen (HBsAg) and/or HBV DNA was positive for more than 6 months; and (3) patients with completed clinical records. Patients with one of the following situations were excluded: (1) patients diagnosed with cholangiocarcinoma, metastases, or other malignancies; (2) complicated with major diseases, such as serious heart, lung, brain, and other important organ dysfunction; and (3) patients infected with HIV, HAV, HDV, HEV, HCV, or other viruses.

The study received approval from the Ethics Committee of The First People’s Hospital of Neijiang (approval no: 2013-055; date: September 2013), and written informed consent was obtained from all participants. The clinical and laboratory information is summarized in Table 1.

### Sample Collection and Follow-Up Survey

Tissue samples were collected in pairs and confirmed by at least 2 pathologists, stored at −80°C.

Patients were followed up for a period ranging from 3 to 60 months to track their recovery and progression. The recurrence, metastasis, and deaths related to HBV-induced HCC were considered the endpoints. Kaplan–Meier and Cox regression analyses were employed to evaluate the follow-up data.

### Cell Culture

HBV-related HCC cell lines (Hep3B2.1-7, HCCLM3, FOCUS, and MHCC97H, ATCC, USA) and a normal liver cell line (THLE-2, ATCC, USA) were maintained in the 10% FBS-containing DMEM culture medium. Cell cultures were kept at 37°C until the cell fusion reached 80-90%.

### Cell Transfection

Cultured cells were transfected with POLR2J4 siRNA (si-POLR2J4) or a negative control (NC) provided by Shanghai GenePharma Co. Ltd. For the co-regulation of POLR2J4 and miR-214-3p, co-transfection of si-POLR2J4 and miR-214-3p inhibitor or inhibitor NC was conducted. Cell transfection was carried out using Lipofectamine 2000 (Invitrogen, Carlsbab, CA, USA) and was estimated by the expression in the cells.

### Real-time Quantitative Polymerase Chain Reaction

Total RNA was isolated using the TransZol Up Plus RNA Kit (Transgen, Beijing, China) and evaluated by the value of A260/A280 (the ratio ranging from 1.8 to 2.2 indicated high quality). cDNA was generated from total RNA with the RevertAid First Strand cDNA Synthesis Kit (Thermo Scientific, Waltham, MA, USA). Then, cDNA was amplified with SYBR Green Mix on the CFX96 PCR System (Bio-Rad, California, CA, USA). The thermal cycles were 95°C for 3 minutes, 40 cycles at 95°C for 12 seconds, and 62°C for 40 seconds. The relative expression was calculated with the 2^−ΔΔCt^ method. GAPDH and U6 were used as the internal standards for POLR2J4 and miR-214-3p, respectively. The primer sequences were: POL2J4 Forward: 5’-AACATCAGGGGAGTTGGGAG-3’, Reverse: 5’-CTTGCACTTTGGGCTTAGCA-3’; GAPDH Forward: 5’-TATGATGATATCAAGAGGGTAGT-3’; Reverse: 5’-TGTATCCAAACTCATTGTCATAC-3’; miR-214-3p Forward: 5’-GCGACAGCAGGCACAGACA-3’; Reverse: 5’-AGTGCAGGGTCCGAGGTATT-3’; U6 Forward: 5’-CTCGCTTCGGCAGCACA-3’; Reverse: 5’-AACGCTTCACGAAATTTGCGT-3’.

### CCK8 Cell Proliferation Assay

Cells were configured into the cell suspension and seeded into the 96-well plates (10^4^ cells/well) with 6 repeated wells per treatment. The CCK8 reagent (10 μL/well) was added at certain time points. The incubation was terminated after incubating for 4 hours with CCK8. OD450 was detected to assess cell proliferation.

### Transwell Migration and Invasion Assay

Cells (5 × 10^4^ cells per well) were seeded into the upper chamber of 24-well Transwell plates maintained with FBS-free culture medium. The lower chamber was filled with complete culture medium. After incubating at 37°C for 1 day, cells on the subsurface of the upper chamber were fixed and viewed under an inverted microscope with a magnification of 200×.

### Luciferase Reporter Assay

The binding sites between POLR2J4 and miR-214-3p were predicted using the online database (shown in Figure 3) for the establishment of wild-type and mutant-type POLR2J4 vectors with the pGL3 plasmids (Madison, CA, Promega, USA). Co-transfection of established vectors and miR-214-3p mimic or inhibitors was performed. The DualGlo luciferase assay system (Promega, Madison, Wisconsin, USA) was employed to analyze POLR2J4 luciferase activity with Renilla as an internal reference.

### Statistical Analysis

All data were presented as mean ± SD and analyzed with SPSS version 26.0 (IBM SPSS Corp.; Armonk, NY, USA) and GraphPad Prism 9.0 software (GraphPad, Boston, MA, USA). The cell experiments were performed in triplicate biological repeats with 3 independent analyses of each. The difference comparison was estimated by the Student’s *t*-test or 1-way ANOVA (*P* < .05). The correlation of POLR2J4 with patients’ features was estimated with the Chi-square test.

## Results

### POLR2J4 was Upregulated in Hepatitis B Virus-Related Hepatocellular Carcinoma

**Figure 1.**
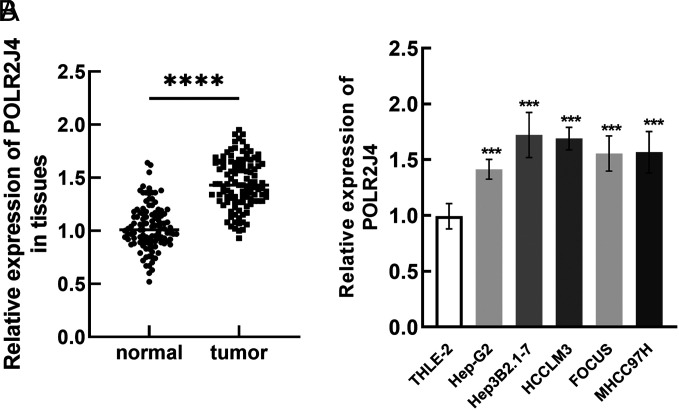
POLR2J4 was upregulated in HBV–HCC tissues (A) and cells (B) compared with corresponding normal tissues and cells. ^****^ *P* < .0001.

In the tissue samples, POLR2J4 was significantly upregulated in the tumor tissues (*P* < .001, Figure 1A). Similarly, in the HBV–HCC cells, elevated levels of POLR2J4 were observed relative to the THLE-2 cell (a normal cell line) and Hep-G2 (a non-HBV cell) (Figure 1B).

### POLR2J4 could Predict Disease Development and Patients’ Outcomes

Study subjects were grouped based on the average POLR2J4 level in their tumor tissues. The low-POLR2J4 group comprised 49 patients with POLR2J4 expression less than 1.75 (mean tissue POLR2J4 level). Meanwhile, the high-POLR2J4 group included 60 patients with POLR2J4 expression over 1.75. A higher POLR2J4 level was closely associated with the presence of cirrhosis (*P *= .029), higher AFP (*P* < .001), advanced Edmondson grade (*P* = .034), TNM stage (*P* = .031), and the presence of vascular invasion (*P* = .022) in HBV–HCC patients (Table 1).

**Figure 2.**
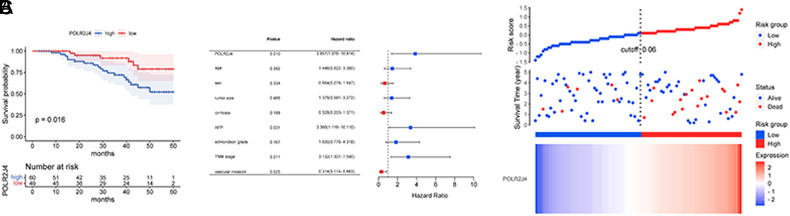
Correlation of POLR2J4 with HBV–HCC patients’ outcomes. (A) Patients with high POLR2J4 expression showed a shorter survival period compared to patients with low POLR2J4. (B) POLR2J4 was identified as an independent prognostic factor together with AFP, TNM, and vascular invasion. (C) Risk score increased with the increasing expression of POLR2J4.

Patients with higher POLR2J4 levels had a lower overall survival rate than those in the low-POLR2J4 group (log-rank *P* = .016, Figure 2A). Moreover, POLR2J4 (HR = 3.857) was identified as an independent adverse factor together with AFP (HR = 3.360), TNM stage (HR = 3.132), and vascular invasion (HR = 0.314, Figure 2B). The risk score model indicated that survival decreased as the risk score increased, which was positively correlated with POLR2J4 levels (Figure 2C).

### POLR2J4 Regulated the Biological Functions of Hepatitis B Virus-Related Hepatocellular Carcinoma Cells via Modulating miR-214-3p

**Figure 3.**
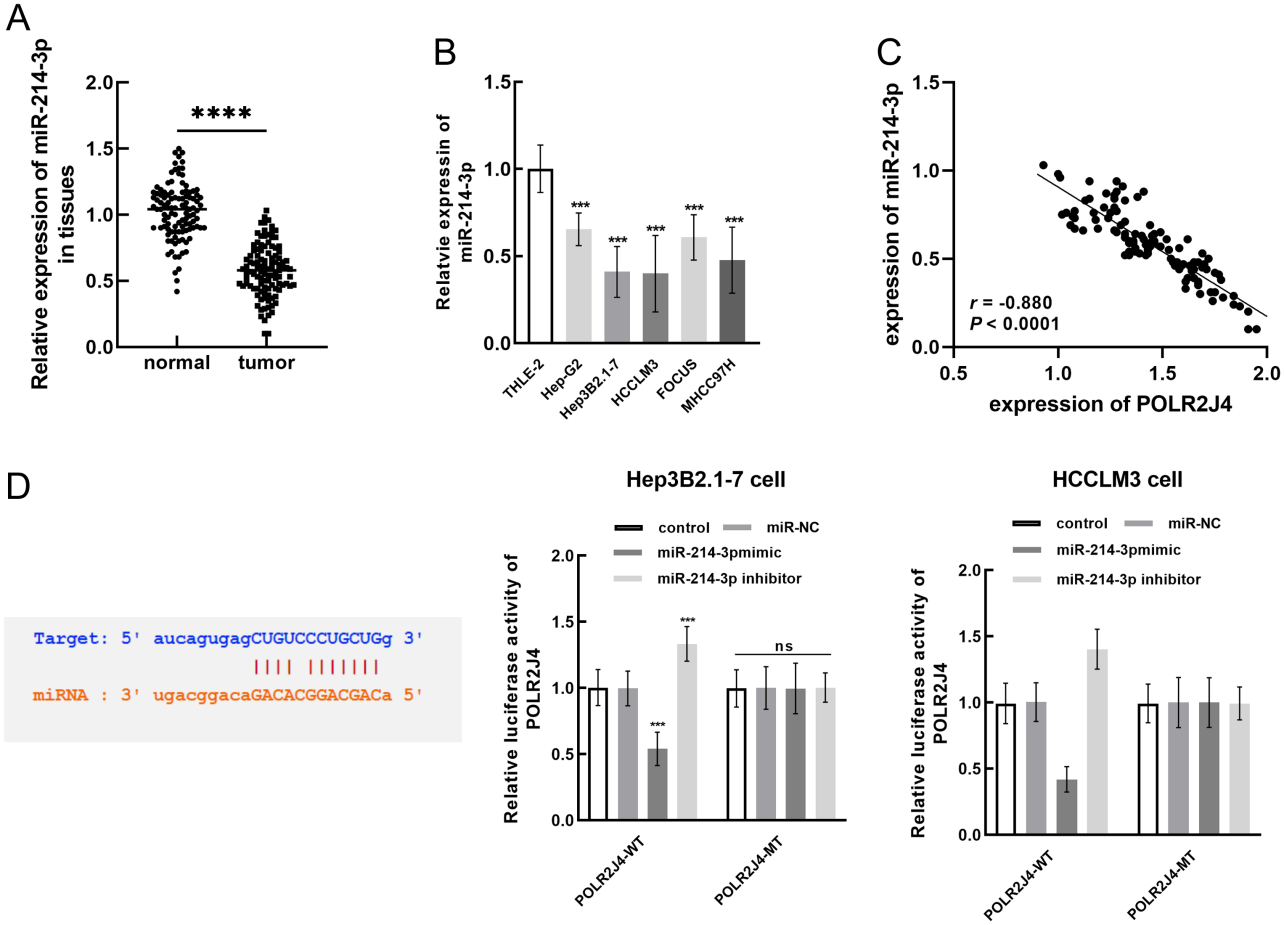
miR-214-3p was downregulated in HBV-HCC tissues (A) and cells (B) and showed a significantly negative correlation with tissue expression of POLR2J4 (C). (D) miR-214-3p negatively regulated the luciferase activity of POLR2J4 with several binding sites predicted from the online database. ^***^P < .001, ^****^P < .0001.

miR-214-3p, predicted to bind with POLR2J4, showed significant downregulation in HBV–HCC tumor tissues (Figure 3A) and cells (Figure 3B) compared to normal tissues, cells, and non-HBV cells (*P* < .001). There was a significant negative correlation between the expression of miR-214-3p and POLR2J4 (*r* = −0.880, Figure 3C). Hep3B2.1-7 and HCCLM3 cells showed relatively high sensitivity to the abnormal expression of both POLR2J4 and miR-214-3p; therefore, the following cell experiments were performed with these 2 cell lines. Luciferase reporter assays indicated the negative regulation of POLR2J4 luciferase activity by miR-214-3p in both Hep3B2.1-7 and HCCLM3 cells (Figure 3D) cells (*P* < .001).

**Figure 4.**
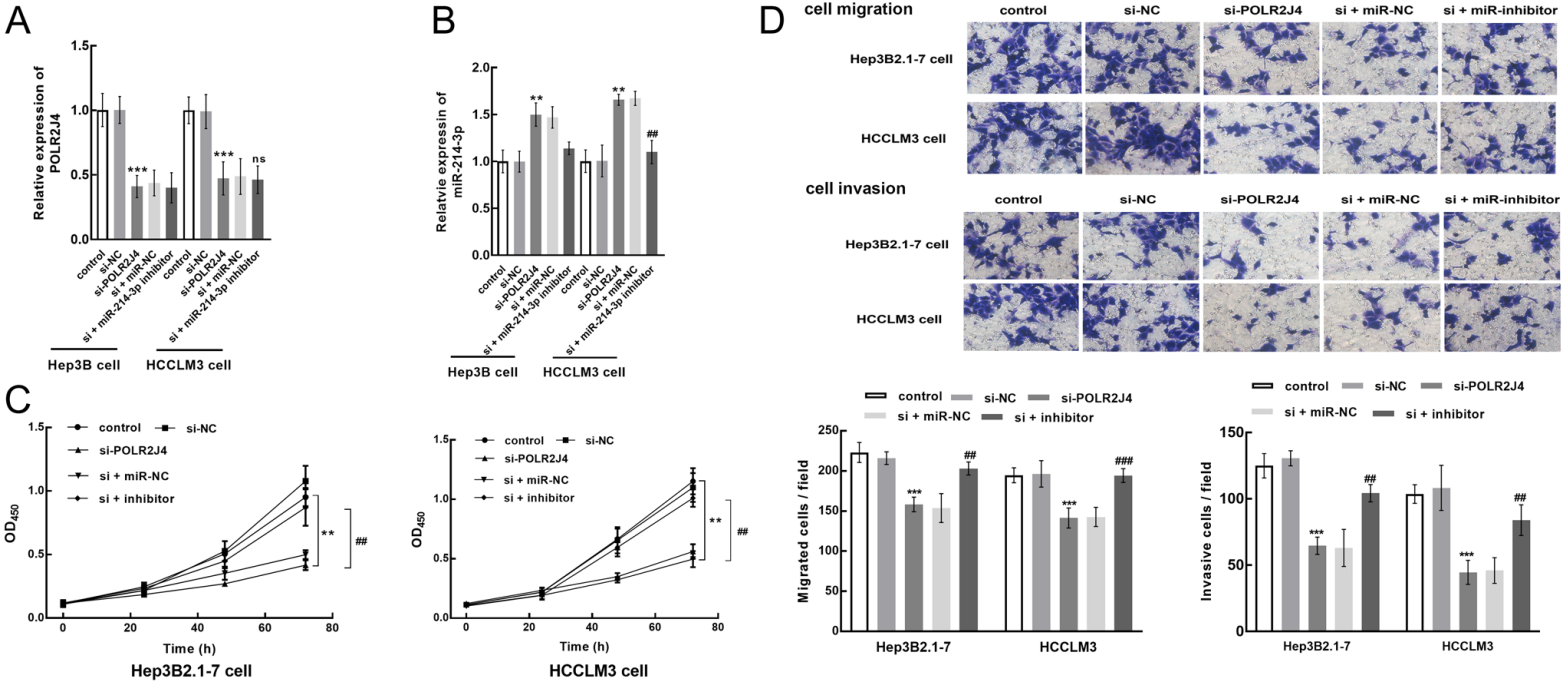
Effect of POLR2J4/miR-214-3p on the cellular processes of HBV–HCC. (A-B) miR-214-3p was enhanced by the knockdown of POLR2J4 but showed insignificant effect on POLR2J4. (C-D) Silencing POLR2J4 suppressed the proliferation (C) and motility ability (D) of HBV–HCC cells, which was attenuated by the suppression of miR-214-3p. ^**^P < .01, ^***^P < .001 relative to the control; ^##^*P* < .01, ^###^*P* < .001 relative to the si-POLR2J4 group.

Transfection of si-POLR2J4 suppressed POLR2J4, and its co-transfection with miR-214-3p inhibitor showed no significant effect (Figure 4A). Notably, si-POLR2J4 enhanced miR-214-3p, which was reversed by the miR-214-3p inhibitor (Figure 4B). The knockdown of POLR2J4 dramatically suppressed both the proliferation (Figure 4C) and motility (Figure 4D) of HBV–HCC cells (*P* < .01). However, the recovery of miR-214-3p could alleviate this inhibition (*P* < .01).

## Discussion

The incidence and mortality rates of HBV–HCC, particularly HBV-induced HCC, remain high. This poses a significant financial burden on society and families.^1,21^ Previously, abnormally expressed lncRNAs have been shown to signal the onset and development of malignant tumors, potentially improving therapeutic efficacy and patients’ outcomes.^22^ Although there is limited data on the specific function and importance of POLR2J4, several lncRNA signatures have highlighted its significance in predicting patients’ prognosis, especially in HCC. POLR2J4, along with the other dysregulated lncRNAs, could predict the overall survival and recurrence-free survival in HCC patients.^13,14,23^ An upregulation of POLR2J4 has been observed in both HBV–HCC tissues and cells. Increased POLR2J4 showed a significant relationship with the severity of patients’ conditions, such as the presence of cirrhosis and vascular invasion, increased AFP, advanced Edmondson grade, and TNM stage. Therefore, POLR2J4 was speculated to be involved in the development of HBV–HCC.

Following the grouping criteria of patients, the overall survival of HBV–HCC patients was compared and associated with POLR2J4. High POLR2J4 levels predict worse outcomes and indicate increasing risk for HBV–HCC patients. POLR2J4 was also identified as an independent prognostic factor alongside traditional indicators, such as AFP, TNM, and vascular invasion, which are consistent with previous reports.^13,14,23^ The Edmondson grade is also a basic pathological classification criterion to assess patients’ conditions and disease severity.^24-26^ However, the prognostic value of the Edmondson grade was insignificant in the present study, which might result from the relatively small sample size. Therefore, further studies with larger sample sizes are of great necessity. Additionally, the present study focused on comparing the expression of POLR2J4 in tumor lesions and adjacent normal tissues. A significant difference was also observed in the expression of POLR2J4 between HBV–HCC cells and non-HBV HCC cells, implying specificity in HBV–HCC. Including a group of control individuals with other etiologies of HCC or liver diseases would help interpret the significance of POLR2J4 more deeply.

Previously, it was shown that Homo sapiens of POLR2J4, circ_007993 acts as a tumor promoter in colorectal cancer by promoting cell proliferation via the miR-203a-3p/CREB1 axis.^27^ Significant upregulation of POLR2J4 was also observed in HBV–HCC cells. Silencing POLR2J4 was found to suppress cell growth, migration, and invasion, suggesting that POLR2J4 could serve as a tumor promoter in the progression of HBV–HCC. LncRNAs could regulate a series of functional genes to mediate the progression of HBV-induced HCC. POLR2J4 and miR-214-3p were predicted to bind with each other, and a significant negative correlation was observed between the expression of POLR2J4 and miR-214-3p in HBV-induced HCC tumor tissues. The dysregulation of miR-214-3p has been widely reported in human cancers and has also been considered a critical tumor regulator. For example, the downregulation of miR-214-3p in retinoblastoma mediates tumor progression and regulates the drug resistance of tumor cells.^28^ In HCC, miR-214-3p was suggested to suppress cell proliferation and cycle and showed a significant correlation with clinical prognosis.^15^ Moreover, miR-214-3p could mediate the induced effect of ketamine on ferroptosis of HCC and has been disclosed to mediate the function of lncRNA LINC00665, BACE-AS, HOXA11-AS, and several other lncRNAs in HCC development.^16-20,29^ Herein, miR-214-3p was negatively regulated by POLR2J4. In addition, although miR-214-3p could not regulate the expression of POLR2J4, it significantly reversed the inhibition of HBV–HCC cell growth and metastasis by POLR2J4 knockdown.

The function of lncRNAs may vary based on their subcellular localization, which could provide insight into the regulatory mechanisms governing their functions. If located in the nucleus, lncRNAs could regulate the expression of nuclear factors at the transcript level. Conversely, cytoplasmic localization suggests lncRNAs might have post-transcriptional regulatory effects.^30-32^ Therefore, the subcellular location of POLR2J4 requires further validation to clarify the molecular mechanism and provide direction for future investigations. POLR2J4 was selected based on previous studies focused on expression profiles in HCC, which is a commonly used and effective means for screening candidate biomarkers. Hence, this research and the investigative methods can be referred in future studies to explore more biomarkers for HCC, benefiting its clinical management. On the other hand, *in vivo* validation is necessary for further investigations, which could improve the persuasiveness of the findings and provide more evidence of reference values for clinical practice.

In conclusion, upregulated POLR2J4 in HBV–HCC could predict the malignant progression and unfavorable prognosis in patients. The knockdown of POLR2J4 hindered HBV–HCC cell growth and motility, an effect which was mitigated by miR-214-3p. Thus, POLR2J4 could act as both a prognostic biomarker and a tumor promoter by regulating miR-214-3p.

## Figures and Tables

**Table 1.** Association of POLR2J4 with Patients’ Clinical Features

	Cases (n = 109)	POLR2J4 Expression		*P*
		Low (n = 49)	High (n = 60)	
Age (years)				.750
≤55	53	23	30	
>55	56	26	30	
Sex				.658
Male	67	29	38	
Female	42	20	22	
Tumor size (cm)				.151
≤5	54	28	26	
>5	55	21	34	
Cirrhosis				.029
Present	72	27	45	
Absent	37	22	15	
AFP (μg/L)				<.001
≤20	46	31	15	
>20	63	18	45	
Edmondson grade				.034
I-II	73	38	35	
III-IV	36	11	25	
TNM stage				.031
I-II	68	36	32	
III	41	13	28	
Vascular invasion				.022
Present	37	11	26	
Absent	72	38	34	

AFP, alpha fetoprotein.
